# From access to capability: digital literacy and health inequality in contemporary China

**DOI:** 10.3389/fpubh.2026.1843334

**Published:** 2026-05-20

**Authors:** Guangyu Chen, Jinyuan He

**Affiliations:** 1 Changzhou Administration Institute, Changzhou, China; 2 School of Government, Changzhou University, Changzhou, China; 3 The Second People’s Hospital of Changzhou, the Third Affiliated Hospital of Nanjing Medical University, Changzhou, China

**Keywords:** China, digital inequality, digital literacy, health inequality, internet access, self-rated psychological health

## Abstract

As digital environments become part of everyday life, digital inequality may be increasingly associated with health differences. Using data from the Chinese Social Survey 2023 (CSS2023), this study examines whether digital literacy is associated with self-rated physical health and self-rated psychological health, whether these associations persist beyond traditional socioeconomic status, and how digital literacy among internet users relates to health alongside the broader access divide in the full sample. Ordinary least squares (OLS) models indicate that digital literacy is positively associated with both outcomes after adjustment for age, gender, marriage, hukou (household registration), education, income, and subjective social status. The association is more pronounced for self-rated psychological health than for self-rated physical health. Supplementary models show that internet access is also associated with health in the full sample, while digital literacy captures capability differences among respondents already online. Because these analyses are based on different samples and differently measured variables, the access-literacy comparison is interpreted descriptively rather than as a formal test of relative importance. Heterogeneity analyses suggest that the association with self-rated psychological health is stronger among older respondents and those with lower educational attainment. These findings are consistent with the view that health-related digital inequality extends beyond access and also involves capability differences among internet users. Digital literacy may therefore be understood as a capability-based health-related resource and a dimension of contemporary health inequality, although the cross-sectional design precludes causal inference.

## 1 Introduction

Health inequality has long been central to social science and public health research. Health is not simply a matter of biology or medical care; it is deeply shaped by social stratification and resource distribution (1, 2). Age, education, income, occupation, and subjective social status matter for health because they shape people’s ability to access information, use services, gain social support, and maintain a sense of control over their lives (3, 4), producing a stable social gradient in self-rated health that cannot be fully explained by lifestyle or work environment (5). In this sense, health inequality is largely a result of unequal distribution of health-related resources.

As digital technology becomes embedded in everyday life, many of these resources are increasingly accessed and organized through digital environments. Searching for health information, booking medical appointments, accessing public services, communicating online, and managing daily tasks all require digital engagement (6, 7). Differences in people’s position within digital environments may therefore translate into broader opportunity gaps with implications for health (8).

Research on digital inequality has accordingly moved beyond its early focus on internet access, device ownership, and basic use (9). While access remains a prerequisite for entering digital environments, it does not guarantee comparable benefits among those already online: inequalities also appear in skills, quality of use, and the ability to convert digital opportunities into actual gains (10–13). For health research, this implies that focusing solely on access may provide an incomplete picture of how digital stratification relates to health disparities. Shifting attention from access to capability may therefore offer a more useful lens for examining health-related digital inequality.

Within this shift from access to capability, digital literacy provides a useful way to capture individuals’ ability to benefit from digital environments. It refers to the capacity to search for, understand, evaluate, express, and use information and tools in digital environments (14, 15), can be conceptualized as a capability-based health-related resource. Existing studies have linked digital skills, internet use, and eHealth-related capabilities to health information access, service use, well-being, and psychological states. Two issues, however, remain insufficiently addressed: digital literacy is often examined within specific digital health contexts rather than situated in the broader framework of health inequality, and less is known about whether general digital literacy is associated with both physical and psychological health beyond traditional socioeconomic status, or whether health-related digital inequality is reflected only in access or also in capability differences among those already online.

China provides an instructive setting for examining these questions. Rapid digital transformation has embedded platform-based services and mobile internet into everyday life, while digital capability and its benefits remain unevenly distributed, especially among older adults (16). National survey evidence also suggests that China’s digital divide is associated with health inequality (17), and that internet development may generate both a “digital dividend” and a “digital divide” rather than automatically reducing health disparities (18).

To examine these issues empirically, this study uses data from the Chinese Social Survey 2023 (CSS2023), a national survey of Chinese adults, and estimates multivariable regression models of self-rated physical and psychological health, adjusting for age, gender, marriage, hukou, education, income, and subjective social status. We also examine internet access in the broader sample and digital literacy among respondents already online as two sequential layers of digital inequality. Because the two measures are observed in different analytic samples, the access–capability comparison is interpreted descriptively rather than as a formal test of relative importance. Given the cross-sectional design, we focus on associations rather than causal effects.

The study contributes to the literature in three ways. Conceptually, it links the resource perspective on health inequality with second- and third-level digital divide research by treating digital literacy as a capability-based health-related resource rather than a technical skill or a form of internet use. Empirically, it provides Chinese national-survey evidence on whether digital literacy is associated with both physical and psychological health beyond conventional stratification factors. Analytically, it distinguishes internet access in the full sample from digital literacy among internet users, treating access and capability as two sequential layers of digital inequality rather than interchangeable measures.

## 2 Literature review

### 2.1 Health inequality and digital inequality: from access to capability

Health differences are embedded in broader systems of social stratification rather than isolated individual outcomes (2). Education, income, occupational status, and subjective social status matter not only because they reflect material living conditions, but because they shape access to information, services, social support, and a sense of control (3, 4). Health inequality can therefore be understood as the unequal distribution and long-term accumulation of health-related resources across social groups, with deeper social conditions and resource allocation mechanisms underlying observed disparities (19). A key implication is that the resources relevant to health are not fixed: as information, services, support, and institutional access increasingly depend on digital systems, new forms of capability may condition how individuals convert digitalized opportunities into usable health-related resources.

Early work on the digital divide focused mainly on internet access, device ownership, and basic opportunities for use (9). This focus remains important, but as access has expanded, digital inequality has increasingly appeared in skills, patterns of use, and the benefits people obtain (10–12, 20). These second- and third-level inequalities matter because they intersect with wider social inequality and shape access to education, employment, services, and participation (8). For health research, the implication is that access alone does not ensure that people can search for information, evaluate content, complete tasks, or gain comparable benefits (13). In digitalized societies, health-related digital inequality may therefore reflect not only a connectivity gap, but also differences in capability.

### 2.2 Digital literacy as a capability-based health-related resource

Within this shift from access to capability, digital literacy provides a useful analytic focus. It refers to the ability to search, understand, evaluate, express, and use information and tools in digital environments (14), and it is conceptually distinct from internet access (entry into digital environments) and from frequency of internet use (exposure or engagement). It is also broader than health-specific eHealth literacy, although the two are related: some studies suggest that digital health literacy may function as a “super determinant of health” because it shapes access to other health-related resources (21). The point relevant here is that digital capability may be associated with how individuals access and mobilize increasingly digitalized resources. Digital literacy is closely related to age, education, and income, but is not reducible to them, as group differences persist in both digital skills and quality of use (12, 22), and it is not fixed, since eHealth-related skills can be improved through training and targeted support (23). This raises the empirical question of whether digital literacy remains associated with health after traditional socioeconomic status is taken into account.

Several pathways may link digital literacy to health. First, stronger digital skills support information access and evaluation. People with higher digital literacy can more easily search for relevant information, compare sources, identify misleading content, and translate online information into practical judgment (14, 24–26). This capacity is especially relevant in health contexts, where online information is abundant but uneven in quality. Second, digital literacy is linked to service use and institutional navigation. Medical appointments, public administration, social security, and other routine services increasingly require digital competence. Access alone does not ensure the skills needed to complete such tasks (22, 27), and the social patterning of eHealth use suggests that digital services may widen opportunities while also reproducing inequality (28–30). Finally, digital environments are important spaces for sustaining family contact, social interaction, and emotional support, so higher digital capability may matter especially for psychological health (8, 31–33). Without adequate support, however, disadvantaged groups may experience frustration, dependence, and exclusion in digital environments, reflecting not only individual limitations but also service design and institutional arrangements (29, 34). The health significance of digital literacy should therefore be understood within the broader context of everyday life and social participation rather than narrowly in terms of medical use alone (21).

### 2.3 Digital inequality and health stratification in the Chinese context

China provides an important setting for examining the relationship between digital literacy and health. Rapid digital transformation has embedded mobile internet, platform economies, and digital services into payment, transport, communication, government services, and healthcare. Research has linked digital infrastructure to public health in China (35), while national survey evidence suggests that China’s digital divide is associated with health inequality (17).

At the same time, digital capability and its returns remain unevenly distributed. Studies show that eHealth behaviors in China remain limited overall and marked by a significant digital divide (36). Internet development may generate both a “digital dividend” and a “digital divide,” rather than automatically reducing health inequality (18). This pattern is particularly visible among vulnerable groups: the digital divide is associated with the health of older migrants (37), digital literacy is systematically related to personal health in national panel data (38), and digital literacy is positively associated with self-rated health, with psychological health as one possible pathway (39).

Taken together, these studies suggest that digital inequality in China is closely intertwined with traditional social stratification and may reflect how existing inequalities are reproduced during digital transformation. Age, education, income, and hukou continue to shape people’s ability to enter and effectively use digital environments. This makes China a useful setting for examining whether digital literacy remains associated with physical and psychological health beyond traditional socioeconomic status, and whether capability differences among those already online represent a further layer of health-related digital inequality.

### 2.4 Research expectations

Based on the above discussion, this study examines four associational research expectations rather than formal causal hypotheses. First, digital literacy is expected to be positively associated with self-rated physical health. Second, digital literacy is expected to be positively associated with self-rated psychological health. Third, these associations are expected to persist after adjustment for traditional stratification factors, including age, education, income, hukou, and subjective social status. Fourth, if capability differences represent a further layer of digital inequality, digital literacy is expected to remain associated with health among internet users, while internet access captures the broader access layer in the full sample.

## 3 Materials and methods

### 3.1 Data source and analytic sample

This study uses data from the Chinese Social Survey 2023 (CSS2023), a national survey of Chinese adults covering demographic characteristics, social attitudes, social participation, digital life, and health. It provides suitable data for examining the relationship between digital inequality and health inequality.

The main analysis focuses on digital literacy and health. The smaller sample size for the digital literacy models mainly reflects the CSS2023 questionnaire design. Respondents were first asked whether they usually used the internet. Those who reported not using the internet skipped the subsequent internet-use and digital literacy questions. In addition, the eight digital literacy items were included only in the random Form A module of the questionnaire. Therefore, digital literacy was observed only for respondents who both used the internet and were assigned to the relevant questionnaire module. Responses coded as “hard to say” were treated as missing. Additional case loss resulted from missing values on health outcomes or covariates.

Because missingness differs slightly across outcomes, the analytic sample includes 4,688 cases for self-rated physical health and 4,671 cases for self-rated psychological health. Supplementary models using internet access rely on a larger sample, with 12,345 cases for self-rated physical health and 12,275 cases for self-rated psychological health. The internet access models therefore capture the access layer of digital inequality in the broader sample, whereas the digital literacy models capture capability differences among internet users with valid digital literacy information in the Form A module.

To assess sensitivity to sample composition and missing-data patterns, we also construct a unified complete-case sample with no missing values on both health outcomes, digital literacy, and all covariates (*N* = 4,665) and re-estimate the fully adjusted models. Except for robustness checks, models are estimated using available cases. We additionally compare respondents included in the digital literacy analytic sample with those excluded from it in Appendix Table A8 to provide a descriptive assessment of possible sample selection.

### 3.2 Measures

#### 3.2.1 Dependent variables: physical health and psychological health

We use two self-rated health measures from CSS2023 to capture physical health and psychological health. The original survey question asks: “Overall, how would you rate your current health status? Please use a score from 1 to 10, where 1 means very poor and 10 means very good.” Respondents then rated two dimensions: “current physical health status” and “current psychological health status.” Both items are measured on 1–10 scales, with higher scores indicating better perceived health. Responses coded as “hard to say” were treated as missing.

The psychological health item should be interpreted as self-rated psychological health, or general self-perceived psychological well-being, rather than as a clinical diagnosis of mental disorder or a validated multi-item mental health scale. For readability, we refer to these two self-rated 1–10 measures as “physical health” and “psychological health” hereafter. Although self-rated health measures are subjective, they are widely used in population health research and show stable associations with actual health status and subsequent health outcomes (40, 41).

In the main analysis, both outcomes are treated as approximately continuous and estimated using ordinary least squares (OLS). Because the original responses are ordered categories, we also re-estimate the fully adjusted models using ordered logit as a robustness check. In applied research, linear models for multi-point scales are common and usually do not materially affect substantive conclusions (42).

#### 3.2.2 Key independent variable: digital literacy

The key independent variable is digital literacy, constructed from eight CSS2023 items in the random Form A module. These items were asked after respondents reported internet use and therefore capture digital capability among respondents who had entered digital environments. These items ask whether respondents can use search engines to search for information, shop online using a mobile phone or computer, are willing to try new apps, read terms of use or privacy policies before downloading apps, recognize that different viewpoints exist online, know how to verify the source and authenticity of online information, know how to express opinions on social media platforms, and can create and post photo or video content online. Response options range from 1 = very inconsistent to 4 = very consistent; responses coded as “hard to say” were treated as missing (Appendix Table A1).

Conceptually, these items capture several dimensions of general digital capability, including information search, platform-based task completion, adaptive use, privacy awareness, information evaluation, online expression, and content production. The measure therefore goes beyond simple device operation or internet access, and it is distinct from frequency of internet use because the items focus on perceived capability, knowledge, and task completion rather than how often respondents go online. At the same time, it should be interpreted as general digital literacy rather than health-specific eHealth literacy.

We combine the eight items into a single averaged index, with higher values indicating stronger digital literacy. This choice is consistent with the study’s focus on general digital capability rather than separate subskills. The scale has high internal consistency (Cronbach’s alpha = 0.882). An exploratory factor analysis using principal axis factoring further supported the use of a single composite index: the Kaiser–Meyer–Olkin value was 0.903, Bartlett’s test of sphericity was significant (*p* < 0.001), only one factor had an eigenvalue greater than 1, and all factor loadings were above 0.50, ranging from 0.562 to 0.787. Corrected item-total correlations ranged from 0.528 to 0.727. Averaging the items preserves the original 1–4 metric. For robustness, we also standardize digital literacy as a z-score and re-estimate the models (Appendix Table A1).

#### 3.2.3 Supplementary independent variable: internet access

To examine the access layer of digital inequality, supplementary analyses use internet access, coded 1 = yes and 0 = no. Unlike digital literacy, internet access indicates whether respondents have entered the digital environment, not how well they can use it.

Because the digital literacy items were asked only of internet users assigned to the random Form A module, digital literacy captures capability differences among connected respondents with valid literacy information, not in the full population. Once the analysis is limited to respondents with valid digital literacy data, internet access no longer varies and cannot be estimated alongside digital literacy in the same sample. We therefore treat internet access and digital literacy as two related but distinct dimensions of digital inequality: the former captures entry into digital environments, and the latter captures capability differences within them. The two measures are not interpreted as directly competing predictors in the same analytic population.

#### 3.2.4 Control variables

To assess whether digital literacy is associated with health beyond traditional socioeconomic status, we include age, gender, marriage, hukou, education, the natural log of annual personal income, and subjective social status. Age is measured in years. Gender is coded as 1 = male and 2 = female. Marriage is coded as 1 = currently partnered and 0 = not currently partnered; the partnered category includes married, remarried, and cohabiting respondents, while the not partnered category includes never married, divorced, and widowed respondents. Hukou is coded as 1 = agricultural hukou and 2 = non-agricultural hukou. Education is coded as an ordinal variable ranging from 1 to 9, with higher values indicating higher educational attainment. Annual personal income is log-transformed to reduce skewness. Subjective social status is recoded from 1 = lowest to 5 = highest, with higher scores indicating a higher perceived social position. These variables account for conventional stratification factors in health inequality research and allow us to assess whether digital literacy shows an association beyond traditional socioeconomic status.

### 3.3 Analytical strategy

We begin with descriptive statistics and an assessment of the internal consistency of the digital literacy scale. We then examine bivariate correlations between digital literacy, health outcomes, and key stratification variables.

The main analysis estimates the relationship between digital literacy and health using stepwise OLS models for physical health and psychological health separately. Model 1 (M1) includes digital literacy only. Model 2 (M2) adds age, gender, marriage, and hukou. Model 3 (M3) further adds education and logged personal income. Model 4 (M4) additionally includes subjective social status. This strategy allows us to assess whether digital literacy remains associated with health after adjustment for demographic characteristics, objective socioeconomic status (SES), and subjective social status. Because the digital literacy sample is restricted by internet-use status and random Form A assignment, the main models are interpreted as analyses of capability differences among respondents with valid digital literacy information rather than as estimates for the full adult population.


Healthi=α+β1DigitalLiteracyi+β2Xi+εi

Healthi is measured as physical or psychological health. DigitalLiteracyi is the key independent variable. X denotes the covariates, and εi is the error term.

To examine access and capability as two layers of digital inequality, we estimate parallel stepwise models replacing digital literacy with internet access. The internet access models are based on the larger full sample and capture whether entry into the digital environment is associated with health. The digital literacy models are based on the internet-user subsample and capture whether capability differences among those already online are associated with health. Because digital literacy is measured only among internet users, internet access and digital literacy cannot be estimated side by side in the same analytic sample. We therefore interpret the two sets of models as describing different layers of digital inequality: internet access in the broader population and digital capability among respondents already online. Standardized coefficients are reported to aid interpretation, but we do not treat differences in coefficient size as a formal test that one dimension is more important than the other. Because the two sets of models use different analytic samples and differently measured variables, the comparison is descriptive and should be interpreted with caution.

We also examine heterogeneity by age and education using interaction terms. Age-group models divide respondents into 18–39, 40–59, and 60–69 years, and interact digital literacy with age group. Educational heterogeneity is assessed by adding an interaction between digital literacy and education. These models test whether the association between digital literacy and health varies across age groups or educational levels. We assessed multicollinearity using variance inflation factors (VIFs). In the fully adjusted main models and the education interaction model, all VIF values were below 2.1, indicating no serious multicollinearity; higher VIFs in the age-group interaction models reflected structural collinearity introduced by dummy-coded interaction terms.


$$\begin{array}{l} \text{Healthi}=\alpha +\beta 1\;\text{DigitalLiteracyi}+\beta 2\;\text{AgeGroupi}\\ +\beta 3\;(\text{DigitalLiteracyi}\times \text{AgeGroupi}\;)+\beta 4\;\mathrm{Xi}+\mathrm{\varepsilon i}\; \end{array}$$

$$\begin{array}{l} \text{PsychologicalHealthi}=\alpha +\beta 1\;\text{DigitalLiteracyi}+\beta 2\;\text{Educationi}\\ +\beta 3\;(\text{DigitalLiteracyi}\times \text{Educationi}\;)+\beta 4\;\mathrm{Xi}+\mathrm{\varepsilon i}\; \end{array}$$

As a further sensitivity check, we re-estimated the fully adjusted models using the CSS weight variable and clustering by primary sampling units (PSU) in SPSS Complex Samples.

### 3.4 Robustness checks

We conduct three robustness checks. First, we re-estimate the fully adjusted models using the unified complete-case sample to assess sensitivity to sample composition and missing-data patterns. Second, we re-estimate the fully adjusted models using ordered logit to assess sensitivity to the linear OLS specification. Third, we standardize digital literacy as a z-score and re-estimate the models to assess sensitivity to the original scale metric.

## 4 Results

### 4.1 Descriptive statistics

Table 1 reports descriptive statistics for the main variables. Overall, respondents reported moderately good physical and psychological health. In the full sample, mean self-rated physical health was 7.14 (standard deviation, SD = 2.23) and mean psychological health was 7.89 (SD = 2.14). In the digital literacy module subsample, the composite digital literacy score had a mean of 2.52 (SD = 0.76), indicating substantial variation in digital capability.

**Table 1:** Descriptive statistics of variables used in the analysis.

Variable	Coding/range	Mean	SD	N
Physical health (self-rated)	1–10, higher = better	7.14	2.23	12,910
Psychological health (self-rated)	1–10, higher = better	7.89	2.14	12,823
Digital literacy	Mean of eight items, 1–4, higher = greater digital literacy	2.52	0.76	4,897
Internet access	1 = yes, 0 = no	0.75	0.43	13,035
Age	18–69 years	46.85	14.22	13,035
Marriage	0 = not currently partnered, 1 = currently partnered	0.78	0.42	13,025
Gender	1 = male, 2 = female	1.55	0.50	13,035
Education	1–9, 1 = non educated, 9 = postgraduate	4.00	2.23	13,031
Hukou	1 = agricultural, 2 = non- agricultural	1.36	0.48	12,990
Logged annual personal income	log-transformed	8.58	3.80	12,697
Subjective social status	1–5, higher = higher perceived status	2.18	0.93	12,787
*N* (valid)				4,665

Marriage was recoded into a binary indicator of current partnership (1 = currently partnered, including married, remarried, and cohabiting; 0 = not currently partnered, including never married, divorced, and widowed).

About 75% of respondents reported internet use, suggesting that internet access is widespread but not universal. The mean age was 46.85 years, and education, income, and subjective social status also showed substantial variation. These patterns provide the stratification needed to examine the relationship between digital inequality and health inequality.

Appendix Table A8 compares respondents included in the unified complete-case digital literacy sample with those excluded from it. Included respondents were younger, more educated, more likely to have non-agricultural hukou and internet access, and reported higher logged income, subjective social status, and self-rated physical and psychological health than excluded respondents.

### 4.2 Scale reliability and bivariate correlations

The eight-item digital literacy scale showed high internal consistency, with a Cronbach’s alpha of 0.882 (Appendix Table A1). Corrected item-total correlations ranged from 0.528 to 0.727, and deleting any item did not meaningfully improve reliability. We therefore combined the eight items into a single averaged composite index.

Bivariate correlations show that digital literacy is positively associated with both health outcomes. In the valid digital literacy subsample, the correlation with physical health was r = 0.196 (*p* < 0.001) and the correlation with psychological health was r = 0.146 (*p* < 0.001), indicating that respondents with higher digital literacy tended to report better health, especially physical health.

Digital literacy was also strongly patterned by traditional stratification variables. It was negatively correlated with age (r = −0.598, *p* < 0.001) and positively correlated with education (r = 0.564, *p* < 0.001) in both health samples. It was also positively, though more modestly, related to income and subjective social status. These results suggest both that digital literacy is related to health and that it is embedded in broader social stratification, making multivariable adjustment necessary.

### 4.3 Digital literacy and health: multivariable regression results

Tables 2, 3 shows the stepwise regression results for digital literacy and health. Overall, digital literacy was positively associated with both physical and psychological health, and these associations remained after adjustment for demographic characteristics, conventional SES, and subjective social status.

**Table 2:** Associations of digital literacy with physical health and psychological health (physical health).

Variables	M1	M2	M3	M4
Digital literacy	0.536*** (0.039)	0.218*** (0.049)	0.184*** (0.053)	0.150** (0.052)
Age		−0.035*** (0.003)	−0.037*** (0.003)	−0.040*** (0.003)
Gender		−0.265*** (0.059)	−0.210*** (0.060)	−0.276*** (0.059)
Marriage		0.310*** (0.077)	0.279*** (0.077)	0.245** (0.076)
Hukou		−0.064 (0.061)	−0.076 (0.064)	−0.107 (0.063)
Education			−0.004 (0.017)	−0.031 (0.017)
Ln personal income			0.036*** (0.008)	0.028*** (0.008)
Subjective social status				0.424*** (0.032)
Constant	5.930*** (0.103)	8.492*** (0.234)	8.349*** (0.239)	7.973*** (0.236)
*N*	4,688	4,688	4,688	4,688
R^2^	0.039	0.071	0.075	0.110
Adj. R^2^	0.038	0.070	0.074	0.108

Standardized beta for digital literacy: M1 = 0.196; M2 = 0.080; M3 = 0.067; M4 = 0.055. Entries are unstandardized coefficients, with standard errors in parentheses. * *p* < 0.05, ** *p* < 0.01, *** *p* < 0.001. Gender is coded as 1 = male and 2 = female; hukou is coded as 1 = agricultural and 2 = non-agricultural; education is coded from 1 to 9, with higher values indicating higher educational attainment.

**Table 3:** Associations of digital literacy with physical health and psychological health (psychological health).

Variables	M1	M2	M3	M4
Digital literacy	0.380*** (0.038)	0.362*** (0.048)	0.342*** (0.052)	0.312*** (0.051)
Age		−0.007* (0.003)	−0.009** (0.003)	−0.011*** (0.003)
Gender		−0.187*** (0.057)	−0.150* (0.058)	−0.207*** (0.058)
Marriage		0.360*** (0.075)	0.336*** (0.075)	0.307*** (0.074)
Hukou		−0.003 (0.059)	−0.007 (0.063)	−0.035 (0.062)
Education			−0.007 (0.017)	−0.031 (0.017)
Ln personal income			0.025** (0.008)	0.018* (0.008)
Subjective social status				0.372*** (0.031)
Constant	7.053*** (0.099)	7.417*** (0.228)	7.326*** (0.233)	6.998*** (0.231)
*N*	4,671	4,671	4,671	4,671
R^2^	0.021	0.028	0.030	0.059
Adj. R^2^	0.021	0.027	0.029	0.058

Standardized beta for digital literacy: M1 = 0.146; M2 = 0.140; M3 = 0.132; M4 = 0.120. Entries are unstandardized coefficients, with standard errors in parentheses. * *p* < 0.05, ** *p* < 0.01, *** *p* < 0.001. Gender is coded as 1 = male and 2 = female; Hukou is coded as 1 = agricultural and 2 = non-agricultural; education is coded from 1 to 9, with higher values indicating higher educational attainment.

For physical health, digital literacy was positively associated with self-rated health in the baseline model (M1: B = 0.536, Beta = 0.196, *p* < 0.001). The coefficient declined after adjustment for age, gender, marriage, and hukou (M2: B = 0.218, Beta = 0.080, *p* < 0.001) and remained significant after further adjustment for education and income (M3: B = 0.184, Beta = 0.067, *p* < 0.001). In the fully adjusted model, which additionally included subjective social status, digital literacy remained positively associated with physical health (M4: B = 0.150, Beta = 0.055, *p* = 0.004). Model fit improved from R^2^ = 0.039 in M1 to 0.110 in M4.

For psychological health, the pattern was more stable. Digital literacy was positively associated with psychological health in the baseline model (M1: B = 0.380, Beta = 0.146, *p* < 0.001), and the coefficient changed little after adjustment for demographic variables and hukou (M2: B = 0.362, Beta = 0.140, *p* < 0.001) or for education and income (M3: B = 0.342, Beta = 0.132, *p* < 0.001). In the fully adjusted model, digital literacy remained positively associated with psychological health (M4: B = 0.312, Beta = 0.120, *p* < 0.001). Model fit increased from R^2^ = 0.021 to 0.059.

The standardized coefficients indicate that the association of digital literacy was more robust for psychological than for physical health. In the fully adjusted models, the standardized coefficient was 0.120 for psychological health but 0.055 for physical health. Subjective social status was also positively associated with both outcomes in the fully adjusted models (physical health: B = 0.424, Beta = 0.190, *p* < 0.001; psychological health: B = 0.372, Beta = 0.175, *p* < 0.001). Taken together, these findings show that digital literacy is associated with both physical and psychological health, and that these associations persist after adjustment for age, marriage, hukou, education, income, and subjective social status.

### 4.4 Supplementary analysis: internet access and digital literacy as sequential layers

To distinguish access from capability, Tables 4, 5 re-estimates the models using internet access as the key explanatory variable. Unlike the digital literacy models, which are based on internet users with valid digital literacy data, the internet access models use the larger full sample and capture whether respondents have entered the digital environment.

**Table 4:** Supplementary access-layer models: internet access and physical and psychological health (physical health).

Variables	M1	M2	M3	M4
Internet access	0.807*** (0.046)	0.257*** (0.051)	0.197*** (0.052)	0.160** (0.050)
Age		−0.039*** (0.002)	−0.040*** (0.002)	−0.042*** (0.002)
Gender		−0.337*** (0.039)	−0.253*** (0.040)	−0.322*** (0.039)
Marriage		0.288*** (0.051)	0.256*** (0.051)	0.203*** (0.050)
Hukou		0.038 (0.041)	−0.020 (0.043)	−0.059 (0.042)
Education			0.009 (0.011)	−0.024* (0.011)
Ln personal income			0.050*** (0.005)	0.040*** (0.005)
Subjective social status				0.499*** (0.021)
Constant	6.526*** (0.041)	9.031*** (0.124)	8.630*** (0.142)	8.078*** (0.140)
*N*	12,345	12,345	12,345	12,345
R^2^	0.024	0.073	0.080	0.122
Adj. R^2^	0.024	0.073	0.080	0.122

Standardized beta for internet access: M1 = 0.155; M2 = 0.049; M3 = 0.038; M4 = 0.031. Entries are unstandardized coefficients with standard errors in parentheses. * *p* < 0.05, ** *p* < 0.01, *** *p* < 0.001.

**Table 5:** Supplementary access-layer models: Internet access and physical and psychological health (psychological health).

Variables	M1	M2	M3	M4
Internet access	0.648*** (0.045)	0.391*** (0.050)	0.316*** (0.051)	0.287*** (0.050)
Age		−0.019*** (0.002)	−0.018*** (0.002)	−0.019*** (0.002)
Gender		−0.219*** (0.038)	−0.133*** (0.039)	−0.188*** (0.039)
Marriage		0.386*** (0.050)	0.368*** (0.050)	0.326*** (0.049)
hukou		0.137*** (0.040)	0.046 (0.043)	0.015 (0.042)
Education			0.037*** (0.011)	0.009 (0.011)
Ln personal income			0.046*** (0.005)	0.038*** (0.005)
Subjective social status				0.405*** (0.020)
Constant	7.397*** (0.039)	8.312*** (0.122)	7.788*** (0.139)	7.337*** (0.139)
*N*	12,275	12,275	12,275	12,275
R^2^	0.017	0.031	0.038	0.068
Adj. R^2^	0.017	0.030	0.038	0.068

Standardized beta for internet access: M1 = 0.129; M2 = 0.078; M3 = 0.063; M4 = 0.057. Entries are unstandardized coefficients with standard errors in parentheses. * *p* < 0.05, ** *p* < 0.01, *** *p* < 0.001.

Internet access was positively associated with both health outcomes. For physical health, internet access was positively associated with self-rated health in the baseline model (M1: B = 0.807, Beta = 0.155, *p* < 0.001) and remained significant after full adjustment (M4: B = 0.160, Beta = 0.031, *p* = 0.002). For psychological health, internet access was also positively associated with health in the baseline model (M1: B = 0.648, Beta = 0.129, *p* < 0.001) and remained significant in the fully adjusted model (M4: B = 0.287, Beta = 0.057, *p* < 0.001).

These results show that basic digital inclusion is also associated with health. At the same time, the digital literacy models do not simply duplicate the access effect. Because digital literacy was measured only among internet users, it captures capability differences within the connected population rather than access differences in the full population. The two sets of models therefore represent sequential layers of digital inequality rather than direct competitors in the same sample.

Descriptively, the standardized coefficients in the fully adjusted models were larger for digital literacy than for internet access for both physical health (0.055 vs. 0.031) and psychological health (0.120 vs. 0.057). Because the two sets of models are estimated on different analytic samples and use differently measured variables, this comparison should not be interpreted as a formal test of relative importance. Rather, the pattern is consistent with the view that capability differences among those already online represent an additional layer of health-related digital inequality beyond basic access.

### 4.5 Robustness checks

We conducted three robustness checks (Appendix Tables A2–A4). First, using the unified complete-case sample (*N* = 4,665) did not change the findings: digital literacy remained positively associated with physical health (B = 0.156, Beta = 0.057, *p* = 0.003) and psychological health (B = 0.322, Beta = 0.124, *p* < 0.001). Second, ordered logit models yielded the same substantive pattern, with positive associations between digital literacy and both physical health (B = 0.128, OR = 1.137, *p* = 0.007) and psychological health (B = 0.282, OR = 1.326, *p* < 0.001). Third, standardizing digital literacy as a z-score also left the results unchanged (physical health: B = 0.114, Beta = 0.055, *p* = 0.004; psychological health: B = 0.237, Beta = 0.120, *p* < 0.001). Overall, the main findings were robust to sample definition, model specification, and scale metric.

We also re-estimated the fully adjusted models using the CSS weight variable and clustering by primary sampling units. The coefficient for digital literacy remained positive and statistically significant for both physical health (B = 0.147, *p* = 0.006) and psychological health (B = 0.284, *p* < 0.001), indicating that the main findings were substantively unchanged.

### 4.6 Age heterogeneity analysis

We next examined whether the association between digital literacy and health varied by age, using interaction models for respondents aged 18–39, 40–59, and 60–69 (Appendix Table A5).

For physical health, the main effect of digital literacy remained positive (B = 0.245, *p* = 0.006), but the interactions with the middle-aged and older groups were not significant (digital literacy × middle-aged: B = 0.039, *p* = 0.713; digital literacy × older: B = −0.062, *p* = 0.664). This indicates no meaningful age heterogeneity in the association with physical health.

For psychological health, the pattern differed. The interaction between digital literacy and the older group was positive and significant (B = 0.296, *p* = 0.033), and the interaction with the middle-aged group was also positive, though only marginally significant (B = 0.190, *p* = 0.064). Stratified fully adjusted models showed the same pattern, with stronger coefficients for digital literacy among middle-aged and older adults than among younger adults (Appendix Table A6). These findings suggest that the association between digital literacy and psychological health is stronger in later life, whereas the association with physical health shows no clear age heterogeneity.

### 4.7 Educational heterogeneity analysis

We also tested whether the association between digital literacy and self-rated psychological health varied by education by adding a digital literacy × education interaction term to the fully adjusted model (Appendix Table A7). The interaction was negative and significant (B = −0.052, *p* = 0.007), indicating that the positive association between digital literacy and psychological health weakens as education increases. In other words, digital literacy appears more strongly associated with psychological health among respondents with lower educational attainment. In the same model, the main effect of digital literacy remained positive and significant (B = 0.300, *p* < 0.001), whereas the main effect of education was not significant (B = −0.017, *p* = 0.332).

Taken together, the heterogeneity analyses suggest that the association between digital literacy and self-rated psychological health is especially pronounced among older respondents and those with lower educational attainment. This pattern is consistent with the view that digital capability may be a scarcer and potentially compensatory resource in socially disadvantaged groups, a point we return to in the Discussion.

## 5 Discussion

### 5.1 Discussion and implications

The first contribution of this study is to locate digital literacy within a resource-based framework of health inequality. The results show that digital literacy is positively associated with both physical health and psychological health after adjustment for age, gender, marriage, hukou, education, income, and subjective social status. This pattern is consistent with the view that, in a digitalized society, digital literacy should not be understood merely as a technical skill or a form of internet use, but as a capability-based health-related resource. Health inequality research has long emphasized the unequal distribution of material, informational, social, and institutional resources. As information access, service use, social support, and institutional navigation become increasingly embedded in digital environments, the ability to turn digital systems into usable resources may become relevant to contemporary health stratification. Digital literacy is therefore not a substitute for traditional socioeconomic resources; rather, it is layered onto existing stratification and may condition how individuals access and mobilize digitalized resources in everyday life.

A second contribution of this study is to distinguish internet access and digital literacy as two sequential layers of health-related digital inequality. Supplementary analyses show that internet access is associated with health in the broader adult sample, indicating that basic digital inclusion remains relevant. The main digital literacy models, by contrast, focus on respondents who were already online and had valid digital literacy data, and therefore capture capability differences within the connected population. Because these analyses are based on different samples and differently measured variables, they should not be interpreted as a formal test that digital literacy is more important than internet access. Rather, the findings suggest that health-related digital inequality includes both an access layer and a capability layer. This interpretation is consistent with wider research showing that digital inequality increasingly involves skills, quality of use, and unequal returns rather than access alone (10, 12, 22). The heterogeneity analyses further suggest that this capability layer is not evenly distributed: its association with psychological health is stronger among older respondents and those with lower educational attainment. Digital inequality therefore should not be viewed as separate from traditional stratification, but as intertwined with existing age and educational inequalities in its association with health outcomes.

It is also notable that the association between digital literacy and psychological health is more robust than that with physical health. This is consistent with recent research from China suggesting that digital literacy may be linked not only to general health, but also to psychological well-being and related mental health outcomes (39). One possible explanation is that digital capability is closely tied to communication, social connectedness, and support networks, all of which may be especially relevant to self-rated psychological health. Reviews suggest that digital technologies may support well-being by reducing isolation and strengthening social ties, especially among older adults (43), and may facilitate emotional and informational support among adults with chronic conditions (44). By contrast, physical health is more deeply shaped by age, chronic disease burden, and long-term material conditions, which may partly absorb the independent association of digital literacy.

Related evidence on digital exclusion points in the same direction. Qualitative work shows that digital exclusion is associated with reduced social connectedness, weakened self-perception, and disempowerment, especially among people with severe mental illness (45). Although this study does not test such mechanisms directly, the findings are consistent with the present results: as digital environments become more deeply embedded in everyday life, limited digital capability may be experienced as psychological strain, frustration, and exclusion.

The heterogeneity findings further suggest why self-rated psychological health may be especially sensitive to digital literacy among older respondents and those with lower educational attainment. For older adults, digital capability may be closely related to maintaining family contact, accessing everyday services, reducing dependence on others, and preserving a sense of autonomy in an increasingly digitalized environment. Limited capability may also be associated with frustration, embarrassment, or exclusion when routine activities such as mobile payment, transport, appointment booking, information search, or public service access require digital tools. For respondents with lower educational attainment, digital literacy may be a scarcer resource and may therefore be more strongly associated with psychological well-being when present. It may help compensate for other disadvantages by improving access to information, social support, and institutional navigation, while also increasing confidence in dealing with digitalized services. These interpretations remain tentative because the present data do not directly measure these pathways, but they help explain why the observed heterogeneity is more evident for self-rated psychological health than for self-rated physical health.

These findings are especially important in China, where platform-based services and mobile internet are deeply integrated into daily life. In this context, insufficient digital capability may be associated with disadvantages in information access, service use, and social participation. Previous studies have already linked China’s digital divide to health inequality and have shown that internet development may produce both a digital dividend and a digital divide (18). Our findings add that digital capability may be an important dimension of contemporary health stratification in China.

The findings also have several policy-relevant but suggestive implications. Because this study is observational and cross-sectional, the results should not be read as direct evidence that digital literacy interventions would improve health. Nevertheless, the observed associations suggest that efforts to address digital inequality may need to consider capability support in addition to access provision, especially for groups among whom the association with psychological health appears stronger, such as older adults and those with lower educational attainment. Existing reviews indicate that digital skills can be improved through training and targeted support, particularly among older adults (23). Such support may be most useful when combined with accessible service design, simplified digital procedures, offline alternatives, and age-friendly assistance. Importantly, the findings should not be interpreted as placing responsibility only on individuals. Older adults’ digital participation depends on capability, opportunity, and motivation (46), while digital exclusion also reflects service design, support systems, and broader institutional arrangements (45). Future intervention studies are needed to assess whether capability-building or inclusive design can reduce health-related digital inequality.

### 5.2 Limitations

Several limitations should be noted:

First, the data are cross-sectional, so the results should be interpreted as associations rather than causal effects. Reverse causality is plausible: respondents with better physical or psychological health may have more energy, cognitive resources, confidence, or social opportunities to acquire and use digital skills. The observed associations may also reflect unmeasured factors, such as cognitive ability, personality, family support, local digital infrastructure, or community resources. Longitudinal data are needed to clarify temporal ordering and assess causal pathways more rigorously.

Second, both physical health and psychological health are self-rated 1–10 measures. Although such measures are widely used in population health research, they may be influenced by reporting styles, cultural norms, individual expectations, personality, and reference-group comparisons. Respondents with similar objective health conditions may therefore rate their health differently. This issue may be particularly relevant for the psychological health item, which captures self-rated psychological health or general self-perceived psychological well-being rather than a clinical assessment of mental disorder. More broadly, because the outcomes are self-rated, the observed associations should be interpreted as associations with subjective health perceptions, which may partly reflect respondents’ broader life evaluations and reference-group comparisons. The associations may be weaker, or differently patterned, if measured against objective health indicators or clinical assessments. This caveat is particularly relevant for the self-rated psychological health outcome, where the gap between subjective rating and clinical diagnosis may be larger than for self-rated physical health. Future research should incorporate objective health indicators, diagnosed conditions, or validated multi-item mental health scales to assess these relationships more comprehensively.

Third, the digital literacy measure captures general digital capability rather than competence in specific health-related settings. It does not directly measure whether respondents searched for health information online, used online appointment systems, accessed electronic health records, engaged in online consultation, or communicated with healthcare providers through digital platforms. The findings should therefore be interpreted as evidence about general digital capability rather than direct evidence about eHealth service use or specific online healthcare behaviors. In addition, the eight items measure self-reported perceived capability rather than directly observed digital behavior. Self-reported digital skills may diverge from actual task performance, especially among groups with limited digital exposure or with social-desirability concerns. The findings should therefore be interpreted as evidence about respondents’ perceived digital capability rather than as objective measures of real-world digital task performance.

Fourth, the comparison between internet access and digital literacy relies on different analytic samples and differently measured variables. It should therefore be interpreted descriptively rather than as a formal test of relative importance. Differences in standardized coefficients may partly reflect sample restriction, the binary versus continuous measurement of the two variables, and other differences in model composition.

In addition, the digital literacy analytic sample is restricted to internet users assigned to the relevant questionnaire module with complete information. As shown in Appendix Table A8, included respondents differ from excluded respondents in age, education, hukou, income, subjective social status, internet access, and self-rated health. Therefore, the digital literacy models should not be generalized to the full adult population, and residual sample-selection bias may remain.

Despite these limitations, the findings are consistent with the view that digital literacy may be an important health-related resource in digital society. Its associations with physical and psychological health persist beyond traditional socioeconomic factors, pointing to the need to understand health-related digital inequality not only in terms of access, but also in terms of capability.

## 6 Conclusion

Using CSS2023 data, this study examined the associations of digital literacy with self-rated physical and self-rated psychological health in China. Digital literacy was positively associated with both outcomes, and these associations persisted after adjustment for age, education, income, hukou, and subjective social status. Supplementary analyses showed that internet access was also associated with health in the full sample, while digital literacy was associated with health among respondents already online. These findings are consistent with the view that internet access and digital literacy represent two related but distinct layers of health-related digital inequality.

Digital literacy may therefore be understood not simply as a technical skill, but as a capability-based health-related resource. In digitalized societies, health-related digital inequality may involve not only whether people can access digital environments, but also whether they can use them to obtain information, services, and support. In the context of China’s ongoing digital transformation, capability differences among internet users may represent an important dimension of contemporary health stratification.

Because the data are cross-sectional, these findings should be interpreted as associational rather than causal. Future research should use longitudinal data and more direct measures of digital health behavior, social support, and service navigation to examine the pathways linking digital capability and health more rigorously.

## Data Availability

Publicly available datasets were analyzed in this study. This data can be found here: http://csqr.cass.cn/project/dataExplore/6406f9d0ba08b693fbfb7334/dataSet/67b6d947b354ed342ca3b979/basic/info.
