# Supplementary material for: From access to capability: digital literacy and health inequality in contemporary China
Source: Front Public Health. 2026 May 20;14:1843334. doi: 10.3389/fpubh.2026.1843334 (PMC13230076; doi:10.3389/fpubh.2026.1843334)
Supplement: Supplementary file 1 [file Data_Sheet_1.pdf]

Appendix

Appendix Table A1. Items, descriptive statistics, and reliability diagnostics for the digital literacy scale

Item	Dimension	Mean	SD	Corrected item-total correlation	Cronbach's alpha if item deleted	Factor loading
I can use search engines (e.g., Baidu, 360) to search for information	Information search	2.85	1.05	0.727	0.858	0.787
I can shop online using a mobile phone or computer	Platform-based task completion	3.01	1.01	0.715	0.860	0.773
I am willing to try new apps	Adaptive use	1.98	0.88	0.568	0.874	0.602
I read terms of use or privacy policies after downloading apps	Privacy awareness	2.32	1.06	0.528	0.879	0.562
I know that there are different viewpoints online	Information awareness	2.84	0.99	0.651	0.866	0.698
I know how to verify the source and authenticity of online information	Information evaluation	2.50	1.02	0.637	0.868	0.679
I know how to express my opinions on social media platforms	Online expression	2.55	1.06	0.715	0.860	0.768
I can create and post photo/video content online	Content production	2.39	1.08	0.640	0.868	0.689

Notes: response options range from 1 = very inconsistent to 4 = very consistent. Factor loadings are from a principal axis factoring analysis extracting one factor. KMO = 0.903; Bartlett's test $p < 0.001$; Cronbach's alpha = 0.882, N = 4665

Appendix Table A2. Robustness checks using a harmonized complete-case sample

Panel A. Physical health

Model	B for digital literacy	SE	Beta	p	N	R^2
M1	0.532	0.039	0.195	<0.001	4665	0.038
M2	0.224	0.050	0.082	<0.001	4665	0.067
M3	0.192	0.053	0.071	<0.001	4665	0.072
M4	0.156	0.052	0.057	0.003	4665	0.107

Panel B. Psychological health

Model	B for digital literacy	SE	Beta	p	N	R^2
M1	0.378	0.038	0.146	<0.001	4665	0.021
M2	0.370	0.048	0.142	<0.001	4665	0.023
M3	0.355	0.052	0.137	<0.001	4665	0.026
M4	0.322	0.051	0.124	<0.001	4665	0.055

Note: All models are estimated on the same complete-case sample (N = 4665). Controls in the fully adjusted model include age, gender, marriage, hukou, education, logged personal income, and subjective social status.

Appendix Table A3. Ordered logit robustness checks

Outcome	Coef. for digital literacy	SE	OR	95% CI for coef.	p	N
Physical health	0.128	0.047	1.137	[0.035, 0.221]	0.007	4688
Psychological health	0.282	0.048	1.326	[0.188, 0.376]	<0.001	4671

Note: Coefficients are from fully adjusted ordered logit models controlling for age, gender, marriage, hukou, education, logged personal income, and subjective social status. Because the proportional odds assumption was not fully met, these models are reported only as robustness checks.

Appendix Table A4. Robustness check using standardized digital literacy

Outcome	B	SE	Beta	95% CI	p	N	R^2
Physical health	0.114	0.039	0.055	[0.036, 0.191]	0.004	4688	0.110
Psychological health	0.237	0.039	0.120	[0.161, 0.312]	<0.001	4671	0.059

Note: Entries are coefficients from fully adjusted OLS models in which digital literacy is standardized as a z-score. Covariates include age, gender, marriage, hukou, education, logged personal income, and subjective social status.

Appendix Table A5. Age heterogeneity in the association between digital literacy and health: interaction models

Panel A. Physical health

Variable	B	SE	Beta	p	95% CI
Digital literacy	0.245	0.089	0.090	0.006	[0.071, 0.420]
Middle-aged (40–59)	-0.687	0.296	-0.166	0.020	[-1.266, -0.108]
Older (60-69)	-0.938	0.337	-0.160	0.005	[-1.599, -0.278]
Digital literacy × Middle-aged	0.039	0.106	0.024	0.713	[-0.168, 0.246]
Digital literacy × Older	-0.062	0.143	-0.021	0.664	[-0.342, 0.218]

N = 4688; R^2 = 0.097

Panel B. Psychological health

Variable	B	SE	Beta	p	95% CI
Digital literacy	0.203	0.087	0.078	0.019	[0.033, 0.373]
Middle-aged (40–59)	-0.620	0.288	-0.158	0.031	[-1.185, -0.056]
Older (60-69)	-0.919	0.327	-0.165	0.005	[-1.561, -0.276]
Digital literacy × Middle-aged	0.190	0.103	0.122	0.064	[-0.011, 0.392]
Digital literacy × Older	0.296	0.139	0.104	0.033	[0.024, 0.568]

N = 4671; R^2 = 0.057

Note: Entries are coefficients from fully adjusted OLS models. The reference age group is 18–39 years. All models control for gender, marriage, hukou, education, logged personal income, and subjective social status.

Appendix Table A6. Stratified fully adjusted models of digital literacy and psychological health by age group

Age group	B for digital literacy	SE	Beta	p	95% CI	N	R^2
18–39	0.206	0.085	0.061	0.015	[0.040, 0.373]	1927	0.040
40–59	0.315	0.075	0.109	<0.001	[0.168, 0.462]	2072	0.078
60-69	0.406	0.132	0.130	0.002	[0.147, 0.666]	672	0.071

Note: Entries are coefficients from fully adjusted OLS models stratified by age group. All models control for gender, marriage, hukou, education, logged personal income, and subjective social status.

Appendix Table A7. Educational heterogeneity in the association between digital literacy and psychological health

Variable	B	SE	Beta	p	95% CI
Digital literacy	0.300	0.051	0.115	<0.001	[0.200, 0.400]
Education	-0.017	0.017	-0.019	0.332	[-0.051, 0.017]
Digital literacy × Education	-0.052	0.019	-0.041	0.007	[-0.090, -0.014]

N = 4671, R^2 = 0.061

Note: Coefficients are from a fully adjusted OLS model of psychological health. Digital literacy and education were mean-centered before estimation. The model controls for age, gender, marriage, hukou, logged personal income, and subjective social status.

Appendix Table A8. Comparison of respondents included in and excluded from the digital literacy analytic sample

Variable	Included sample	Excluded respondents	p-value
Self-rated physical health, mean	7.28	7.05	<0.001
Self-rated psychological health, mean	8.01	7.81	<0.001
Age, mean	43.35	48.80	<0.001
Gender: male, %	46.15	43.66	0.006
Marriage: currently partnered, %	75.80	79.00	<0.001
Non-agricultural hukou, %	40.62	33.95	<0.001
Education, mean	4.52	3.71	<0.001
Subjective social status, mean	2.21	2.16	0.007
Internet access, %	100.00	61.83	<0.001
Logged personal income, mean	8.83	8.43	<0.001

Notes: The included sample refers to respondents in the unified complete-case digital literacy sample. The excluded group includes respondents not included in this sample because of internet-use status, random Form A assignment, “hard to say” responses, or missing values on health outcomes or covariates. For binary variables, means are presented as percentages. Non-agricultural hukou is calculated from the hukou variable coded as 1 = agricultural and 2 = non-agricultural. p-values are from two-sample t-tests. The comparison is descriptive and intended to assess possible sample selection.
